# Cerebral Venous Sinus Thrombosis After BNT162b2 mRNA COVID-19 Vaccination

**DOI:** 10.7759/cureus.18775

**Published:** 2021-10-14

**Authors:** Yoshitaka Yamaguchi, Luna Kimihira, Hikaru Nagasawa, Kyoichi Seo, Manabu Wada

**Affiliations:** 1 Department of Neurology, Yamagata Prefectural Central Hospital, Yamagata, JPN; 2 Department of Neurosurgery, Yamagata Prefectural Central Hospital, Yamagata, JPN

**Keywords:** contrast enhanced magnetic resonance imaging (cemri), mrna vaccine, magnetic resonance venography, covid-19, cerebral venous sinus thrombosis (cvst)

## Abstract

W present a rare case of cerebral venous sinus thrombosis after the BNT162b2 mRNA COVID-19 vaccine. A 61-year-old Japanese man developed a headache 10 days after the first dose of the vaccine. Magnetic resonance venography and contrast-enhanced brain MRI showed thrombosis in the superior sagittal sinus and the right transverse sinus. Anticoagulation with intravenous unfractionated heparin followed by oral warfarin was started. His headache improved, and brain MRI on day 22 showed resolution of thrombus. He was maintained on anticoagulation with warfarin and discharged without any neurological sequelae. This case is presented in the context of the relevant literature.

## Introduction

Cerebral venous sinus thrombosis (CVST) is a rare type of stroke, which accounts for around 0.5-1% of all stroke occurrences [1]. Although the pathophysiology is not fully understood, the risk factors include prothrombotic conditions such as protein C/S deficiency, infections, mechanical trauma, vasculitis, systemic diseases including connective tissue diseases and malignancies, and drugs [1]. Since it can present various clinical symptoms and signs, the diagnosis can be sometimes challenging. However, early diagnosis and prompt initiation of optimizing patient care can improve clinical prognosis in these patients.

As vaccination against coronavirus disease 2019 (COVID-19) is promoted all over the world to overcome the COVID-19 pandemic, an emerging concern about thromboembolic side effects after vaccination has been recognized [2-6]. In April 2021, a case series of CVST and other thrombotic events after virus vector COVID-19 vaccines (ChAdOx1 nCoV-19 [Oxford-AstraZeneca] and Ad26.COV2.S [Johnson & Johnson/Janssen]), frequently associated with thrombocytopenia, have been successively reported [2-6]. This vaccine-associated syndrome, called vaccine-induced immune thrombotic thrombocytopenia (VITT), provokes immune-mediated thrombotic thrombocytopenia via IgG antibodies that recognize platelet factor (PF) 4 and activate platelets through their Fcγ receptors [2-6]. Therefore, the pathogenesis of VITT has been considered similar to that of heparin-induced thrombocytopenia (HIT) [2-6]. Recognition of the emergence of VITT has led to temporary suspicion of the use of such vaccines in European countries.

In contrast to the virus vector COVID-19 vaccines, thromboembolic side effects after vaccination with mRNA-based COVID-19 vaccines (BNT162b2 mRNA [Pfizer-BioNTech] and mRNA-1273 [Moderna]) have been rarely reported, with only a few published case reports of CVST [7-9]. The clinical characteristics of thromboembolism after mRNA-based COVID-19 vaccination have not been well clarified compared to VITT caused by virus vector COVID-19 vaccines. Thus, a rare case of CVST that developed after BNT162b2 mRNA vaccination is reported. The clinical characteristics of CVST patients after mRNA-based COVID-19 vaccination in previous case reports were also investigated.

## Case presentation

**Figure 1:**
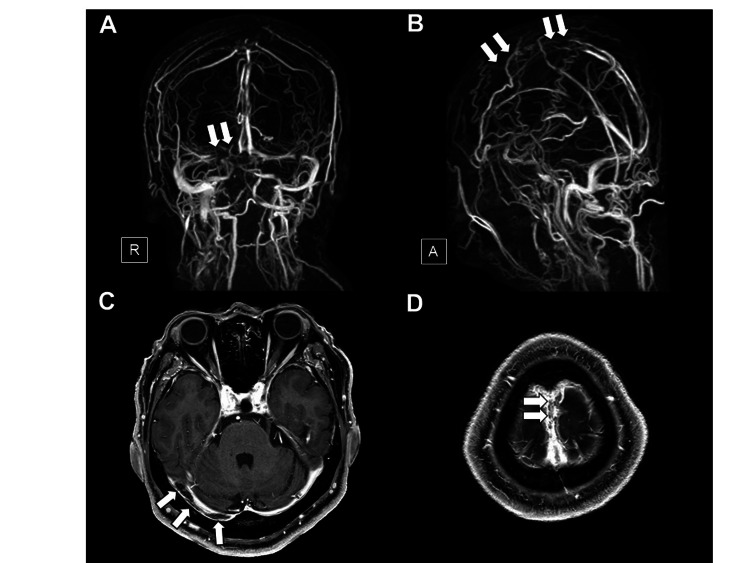
Magnetic resonance venography and brain MRI with gadolinium enhancement on admission Magnetic resonance venography (A, B) and T1-weighted brain MRI with gadolinium enhancement (C, D) on admission show thrombus as a filling defect in the right transverse sinus (A, C) (arrows) and the superior sagittal sinus (B, D) (arrows). R, right; A, anterior; MRI, magnetic resonance imaging

**Figure 2:**
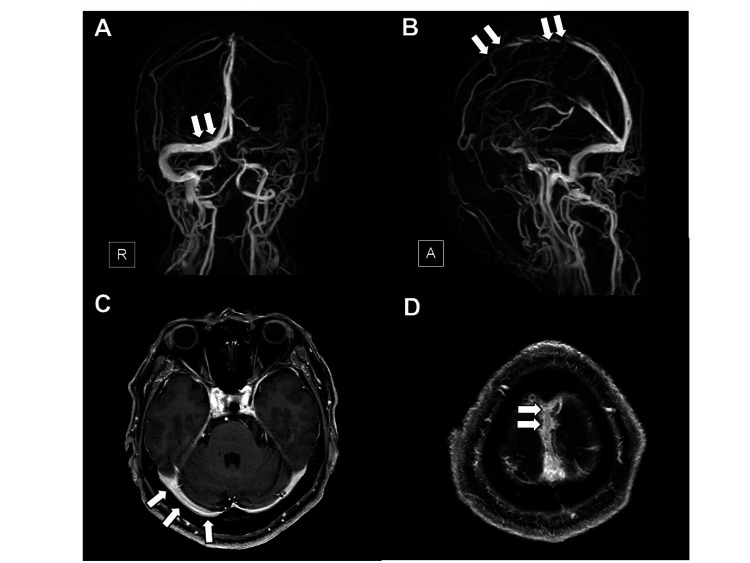
Magnetic resonance venography and brain MRI with gadolinium enhancement on day 22 of admission Magnetic resonance venography (A, B) and T1-weighted brain MRI with gadolinium enhancement (C, D) on day 22 admission show resolution of thrombus in the right transverse sinus (A, C) (arrows) and the superior sagittal sinus (B, D) (arrows). R, right; A, anterior; MRI, magnetic resonance imaging

A 61-year-old Japanese man with a history of hyperuricemia developed a headache 10 days after the first dose of the BNT162b2 mRNA COVID-19 vaccine and was admitted to our hospital two days later. He was afebrile, showed elevated blood pressure (170/104 mmHg), and did not demonstrate any neurological deficits. Although brain magnetic resonance imaging (MRI) showed no abnormalities in the brain parenchyma, including brain ischemic or hemorrhagic lesions, magnetic resonance venography (Figures 1A, 1B) and brain MRI with gadolinium enhancement (Figures 1C, 1D) showed thrombosis in the superior sagittal sinus and the right transverse sinus. His complete blood count was within normal limits, including a normal platelet count (163,000/µL). The level of C-reactive protein was slightly elevated at 1.15 mg/dL (normal: <0.4 mg/dL). Coagulation tests including prothrombin time, activated partial thromboplastin time, fibrinogen, and antithrombin III were normal, except for an elevated D-dimer (3.7 μg/mL; normal: <1.0 μg/mL) level. Serum interleukin (IL)-6 on day 13 after vaccination was elevated at 12.3 pg/mL (normal: <4.0 pg/mL). Prothrombotic examinations for lupus anticoagulant and anti-cardiolipin antibodies were negative, and levels of protein C and protein S were normal. PF 4 antibodies measured by latex agglutination method were negative. Screening for connective tissue diseases and infectious diseases including varicella zoster virus, tuberculosis, and fungal diseases was negative. The polymerase chain reaction test for COVID-19 was negative. Whole-body computed tomography (CT) excluded malignant neoplasms. Thus, no potential diseases associated with the development of CVST were found. Whole-body CT and ultrasound evaluation did not show any other thrombi. He was started on anticoagulation with intravenous unfractionated heparin (UFH) followed by oral administration of warfarin. His headache improved on admission day 13. Brain MRI on day 22 showed resolution of thrombus in the right transverse sinus and the superior sagittal sinus (Figure 2). He was maintained on anticoagulation with warfarin and discharged on admission day 24 without any neurological sequelae. Serum IL-6 level normalized on day 46 after vaccination.

## Discussion

A rare case of CVST that developed after BNT162b2 mRNA COVID-19 vaccination was presented. Because an extensive diagnostic workup did not detect any causative factors for the development of CVST, including coagulopathy, connective tissue diseases, infection, and malignancy, other than vaccination, the present case was diagnosed as CVST associated with BNT162b2 COVID-19 vaccination. Early diagnosis and initiation of anticoagulant therapy resulted in a good outcome. To the best of our knowledge, this is the fourth published case report and also the first Japanese case report of CVST after BNT162b2 COVID-19 vaccination.

No conclusion has been reached about whether mRNA vaccination can be a risk factor for CVST. An American study estimated that the incidence of cerebral venous thrombosis (CVT) after COVID-19 infection was significantly higher than in a matched cohort of people who received an mRNA vaccine (BNT162b2 mRNA or mRNA-1273) (relative risk = 6.33; 95% CI: 1.87-21.40; p = 0.00014) [10]. In addition, 4,047,651 doses of mRNA based vaccine have been administered in Singapore as on May 31, 2021, of which three cases of CVST associated with mRNA based vaccination have been reported, with an incidence of 0.000074% (3/4,047,651) per dose or 0.00017% (3/1,766,497) for people who completed two vaccination doses. The incidence was lower than that of CVT associated with COVID-19 infection, which was 0.0045% (2/44,479) in a local review [9,11]. Data from VigiBase, the WHO global database of individual case safety reports, reported unexpected CVT after vaccination with the ChAdOx1 nCoV-19 vaccine in 1.1% (7/639), with the BNT162b2 mRNA vaccine in 0.4% (4/1,197), and with the mRNA-1273 vaccine in 0.9% (3/325) of events reported [12]. In the Yellow Card report covering the period up to August 25, 2021, the Medicines and Healthcare products Regulatory Agency in the United Kingdom reported 42 CSVT or CVT cases out of a total of 17.3 million second doses of the BNT162b2 mRNA vaccine (0.002428%, 42/17,300,000) [13]. Additionally, a self-controlled case series study from England found an increased risk of CVST after the ChAdOx1 nCoV-19 vaccine (relative risk = 4.01, 95% CI: 2.08 to 7.71 at 8-14 days) and the BNT162b2 mRNA vaccine (relative risk = 3.58, 95% CI: 1.39 to 9.27 at 15-21 days) [14]. On the other hand, a national prospective cohort study in Scotland did not show a positive association between BNT162b2 mRNA vaccination and venous thromboembolic events including CVST [15]. Because the sample size of CVST cases in each study was small, further investigations using large-scale health databases are desirable to establish the risk of CVST following mRNA-based COVID-19 vaccination.

The characteristics of patients in published case reports (seven cases including the present case) who developed CVST after BNT162b2 COVID-19 vaccination are presented in Table 1. All cases were middle-aged (47 to 67 years old), and three of them were female. Of note, four of them developed CVST after the second vaccination, suggesting that the absence of side effects at the first dose does not guarantee freedom from CVST after the second dose. This is consistent with the recent report from Germany that surveyed patients with CVT nationwide through web-based questionnaires [16]. In that report, 45 CVT patients were identified, and all three patients who developed CVT after second vaccination had received the BNT162b2 vaccines [16]. The median number of days from last vaccination to onset was eight days (interquartile range: 3-10 days). Thrombus was located mainly in the superior sagittal sinus, transverse sinus, sigmoid sinus, and jugular veins, and sometimes in the cortical veins. The deep cerebral venous system was not involved. Cerebral venous infarction, intracerebral hemorrhage, and subarachnoid hemorrhage related to CVST were detected in two, three, and three patients, respectively. Three patients did not show any of these parenchymal lesions. In contrast to the characteristic thrombocytopenia seen in VITT patients associated with virus vector COVID-19 vaccines, the platelet count was normal in all cases. Anti-PF 4 antibodies were tested in three cases, and all were negative. All cases were treated with UFH or low-molecular-weight heparin (LMWH) safely followed by warfarin or direct oral anticoagulant treatment. The clinical prognosis was generally good, although two patients required decompression craniectomy due to life-threatening intracerebral hemorrhage [9], and neurological sequelae remained in patients with parenchymal lesions. Based on these characteristics, the clinical profiles of CVST related to mRNA-based vaccination are different from those of VITT patients resembling HIT, and they may be indistinguishable from non-vaccine-related CVST. This is consistent with the recent report from the European Medicines Agency, in which 213 post-vaccination CVST patients (ChAdOx1 nCoV-19 n = 187; BNT162b2 mRNA n = 25; mRNA-1273 n =1) were identified in their database, and thrombocytopenia was not recognized in the mRNA-based vaccine group [17]. The absence of a relationship between mRNA-based vaccines and thrombocytopenia was also consistent with research from Germany [16], England [14], and Scotland [15].

**Table 1:** Clinical characteristics of cases of cerebral venous sinus thrombosis after BNT162b2 vaccination Abbreviations: APX, apixaban; Blt, bilateral; CCMs, cerebral cavernous malformations; CLP, clopidogrel; CV, cortical veins; CVI, cerebral venous infarction; DE, dabigatran etexilate, DM, diabetes mellitus; HL, hyperlipidemia; HT, hypertension; ICH, intracerebral hemorrhage; IDA, iron deficiency anemia; JP, Japan; JV, jugular vein; LMWH, low-molecular-weight heparin; Lt, left; MYS, Malaysia; N.E., not examined; PF4 Ab, anti-platelet factor 4 antibody, PLT, platelet; POR, Portugal; Ref, reference; Rt, right; SAH, subarachnoid hemorrhage; SIN, Singapore; SS, sigmoid sinus; SSS, superior sagittal sinus; TS, transverse sinus, UFH, unfractionated heparin; WF, warfarin

Ref.	Age (years), sex, country	Comorbidities	Symptoms	Days from vaccination to onset	Site of thrombosis	Parenchymal lesions			Laboratory findings			Treatment	Outcome
						CVI	ICH	SAH	PLT (/µL)	PF4 Ab	D-dimer (μg/mL)		
[7]	49, M, MYS	Coronary heart disease	Headache, giddiness	16 (1st dose) (worsened by 2nd dose)	SSS, Lt. TS, Lt. SS, Lt. JV	-	-	-	302,000	N.E.	0.2	LMWH, CLP, APX	Full recovery
[8]	47, F, POR	IDA, adenomyosis, use of oral contraceptives	Headache, hemiparesis, papilledema, nausea, etc.	6 (1st dose)	SSS, Rt. TS, Blt. SS, Rt. JV	+	-	+	343,000	Negative	N.E.	LMWH, WF	Gait disturbance remained
[8]	67, F, POR	CCMs, probable renal cell carcinoma, etc.	Headache, hemiparesis, consciousness disturbance	3 (2nd dose)	CV, SSS, Rt. TS, Rt. SS, Rt. JV	-	-	-	164,000	Negative	N.E.	LMWH, DE	Full recovery
[9]	54, M, SIN	HL	Headache, hemiparesis	1 (2nd dose)	Rt. TS, Rt. SS Rt. JV	-	+	-	300,000	Negative	N.E.	UFH, LMWH, WF	Left hemiparesis remained
[9]	62, F, SIN	HT	Headache, vomiting, behavioral changes	9 (2nd dose)	Rt. TS, Rt. SS Rt. JV	-	+	+	383,000	N.E.	N.E.	UFH, LMWH, WF	Left hemiparesis remained
[9]	60, M, SIN	DM, HT, HL	Giddiness, vomiting, ataxic hemiparesis	8 (2nd dose)	CV, Rt. TS, Rt. SS, Rt. JV	+	+	+	346,000	N.E.	N.E.	LMWH, WF	Full recovery
Our case	61, M, JP	Hyperuricemia	Headache	10 (1st dose)	SSS, Rt. TS, Rt. SS	-	-	-	163,000	Negative	3.7	UFH, WF	Full recovery

The pathophysiological mechanism leading to CVST after mRNA-based vaccination remains unclear. Before translation, the mRNA may bind to pattern recognition receptors inducing pro-inflammatory cascades [18]. The mRNA particles translate a spike protein that may facilitate platelet aggregation and dense granule secretion [19] and activate the alternative pathway [20]. Furthermore, the spike protein in epithelial cells promotes IL-6 trans-signaling by activation of the angiotensin II type 1 receptor axis to initiate coordination of a hyper-inflammatory response [21]. These might contribute to a trigger of thrombus formation, which is quite different from the pathogenesis of VITT mediated by PF4-reactive antibodies [2]. Increased serum IL-6 in the acute phase of CVST in our case may suggest the involvement of hyper-inflammatory response after vaccination.

## Conclusions

A case of CVST after BNT162b2 mRNA COVID-19 vaccination was presented. Previous case reports and the present case suggest that the characteristics of CVST after mRNA-based COVID-19 vaccines are similar to those of non-vaccine-related CVST. Therefore, anticoagulation with UFH or LMWH may be appropriate and should not be avoided in contrast to VITT patients after virus vector COVID-19 vaccination, in which heparinization or platelet transfusion is contraindicated. Although the risk of CVST may be lower after mRNA-based COVID-19 vaccination than after virus vector COVID-19 vaccination or COVID-19 infection, clinicians should note the rare manifestation of CVST in patients with headache or other neurological symptoms due to an unknown cause after mRNA COVID-19 vaccination.
